# The Impact of the *Staphylococcus aureus* Virulome on Infection in a Developing Country: A Cohort Study

**DOI:** 10.3389/fmicb.2017.01662

**Published:** 2017-08-29

**Authors:** Marthe Lebughe, Patrick Phaku, Silke Niemann, Dieudonné Mumba, Georg Peters, Jean-Jacques Muyembe-Tamfum, Alexander Mellmann, Lena Strauß, Frieder Schaumburg

**Affiliations:** ^1^Institut National de Recherche Bio-Médicale, Université de Kinshasa Kinshasa, Democratic Republic of the Congo; ^2^Institute of Medical Microbiology, University Hospital Münster Münster, Germany; ^3^Institute of Hygiene, University Hospital Münster Münster, Germany

**Keywords:** *Staphylococcus aureus*, Africa, whole genome sequencing, virulome, infection

## Abstract

We performed a cohort study to analyze the virulome of *Staphylococcus aureus* from the Democratic Republic of the Congo using whole genome sequencing and to assess its impact on the course of *S. aureus* infections. Community-associated *S. aureus* from nasal colonization (*n* = 100) and infection (*n* = 86) were prospectively collected. Phenotypic susceptibility testing and WGS was done for each isolate. WGS data were used to screen for 79 different virulence factors and for genotyping purposes (*spa* typing, multilocus sequence typing). The majority of the 79 virulence factors were equally distributed among isolates from colonization and infection. Panton-Valentine leukocidin (PVL) and the non-truncated hemolysin β were associated with skin and soft tissue infection (SSTI) and recurrence of disease but did not influence the course of infection (i.e., mortality, surgical intervention). For the first time, we show that not only PVL but also hemolysin β could contribute to the development of SSTI in PVL-endemic areas such as Africa.

## Introduction

Apart from *Salmonella enterica* and *Streptococcus pneumoniae*, *Staphylococcus aureus* is one of the most important pathogen causing invasive infections in sub-Saharan Africa (Reddy et al., 2010). There is some evidence that the incidence of *S. aureus* skin and soft tissue infections (SSTI) is higher in sub-Saharan Africa compared to the United States (Schaumburg et al., 2014a). In fact, *S. aureus* can literally infect almost all kind of tissues causing a broad range of infections (e.g., endocarditis, meningitis, osteomyelitis). Some of these entities are strongly linked to the presence or expression of distinct virulence factors or gene regulators. For instance, the activity of the accessory gene regulator (*agr*) is important for SSTI while *agr* defective isolates were associated with bloodstream infections (Painter et al., 2014). The role of the protein toxin Panton-Valentine leukocidin (PVL) in human disease is still controversial, as epidemiological analyses, *in vitro* studies and animal models show conflicting results which is partly due to the species specific action of PVL on leukocytes (Löffler et al., 2010). However, a recent meta-analysis clearly supports the link between PVL and SSTI (Shallcross et al., 2013). This is of clinical importance as sub-Saharan Africa is considered to be a PVL-endemic region where 17–74% of all *S. aureus* are PVL-positive (Schaumburg et al., 2014b). This high prevalence of PVL among African *S. aureus* also leads to increased anti-PVL antibody levels in Africa (Senegal) compared to Europe (France) (Rasigade et al., 2015). Not only PVL but also hemolysin α has been linked to SSTI which can be effectively prevented by neutralizing antibodies in children (Fritz et al., 2013).

So far, there is only limited knowledge on the virulome of *S. aureus* in Africa using whole genome sequencing (WGS) data. The aims of this study were to describe the virulence factors of the core and accessory genome of *S. aureus* from the Democratic Republic of the Congo and to assess their link with clinical infection in a cohort study.

## Materials and Methods

### Ethics Statement

Ethical clearance was granted by the ethics committee of the University of Kinshasa (ESP/CO/070/2012 and ESP/CO/070b/2014) and the University of Münster (2009-227-b-S). Each participant or its guardian signed a written informed consent prior to enrolment. The authors complied with all of the legal requirements pertaining to the locations in which the work was done.

### Participants

Asymptomatic, community-associated *S. aureus* carriers were recruited in the Kinshasa metropolitan area (e.g., schools, universities, pre-school clinics for vaccination or in the residential neighborhood) between 2013 and 2015. The exclusion criteria for carriers were (i) hospitalization in the past 4 weeks, (ii) antibacterial treatment in the past 4 weeks, and (iii) antituberculous treatment in the past 4 weeks.

Patients with a *S. aureus* infection detected in the microbiology laboratory of the University Hospital Kinshasa were included if positive samples were taken in the out-patient departments or within 48 h after admission to the hospital (including the time spent in a transferring hospital) (Henderson et al., 2013).

Recruitment was stopped either after the detection of 100 consecutive *S. aureus* isolates in each group (colonization/infection) or after a predefined study period of 2.5 years. A sample size of 100 isolates was recently considered to be sufficient for a snapshot of the population structure (Herrmann et al., 2013; Phaku et al., 2016).

A standardized case report form was completed for all participants including demographic data, patient history and comorbidities (incl. McCabe and Charlson score items). Clinical data were obtained from personal health record files. For cases with a *S. aureus* infection, we additionally recorded the site of infection, surgical procedures, antimicrobial therapy and 14 days mortality which is measured to assess the early mortality in clinical trials (Kaasch et al., 2015).

### Bacterial Isolates

Nasal swabs or clinical specimen were cultured on Columbia blood agar. Preliminary species identification of *S. aureus* was based on Gram staining, a positive catalase-, coagulase- and Pastorex-test (Bio-Rad, Marnes-la-Coquette, France). Species confirmation was done in Germany using MALDI-TOF (Bruker, Bremen, Germany). Antimicrobial susceptibility was tested with VITEK2 automated systems (bioMérieux, Marcy l’Etoile, France) and the AST632 test cards.

### Whole Genome Sequencing

Bacterial DNA was extracted from single colony material by mechanical cell lysis (Köser et al., 2014). Shotgun sequencing of 250 bp paired-end libraries aiming for a 100-fold coverage was carried out with NexteraXT chemistry on an Illumina MiSeq or NextSeq sequencing platform (Illumina Inc., San Diego, CA, United States). Quality trimming of FASTQ files (average base quality of 30), *de novo* assembly using Velvet 1.1.04, and bioinformatic genome analysis were performed with SeqSphere^+^ software (version 3.1, Ridom GmbH, Münster, Germany). For quality control, the 1861 cgMLST targets were queried in all genomes (Leopold et al., 2014).

Only genomes harboring ≥ 95% of these core genome targets passed the quality control and were included in the analysis; All FASTQ files were deposited at the ENA database^1^ under the study number PRJEB15192.

The *S. aureus* genomes were screened for the presence and allelic variants of 79 virulence factors (Supplementary Table S1). For each gene, a BLAST search was done against all reference alleles (Strauss et al., 2016). All genes that were found with ≥ 95% nucleotide identity and ≥ 99% length overlap to any reference allele were included. Targets that were partially identified on a cropped contig or “failed targets” (i.e., containing internal stop codons, frame shifts, or nucleotide ambiguities) were only included if they were found within operons/prophages and if the other genes of the respective operon/prophage were present (e.g., *agr*, ACME, *hlg*) (Strauss et al., 2016).

The selection of virulence factors was based on the virulence factors which are included in the Identibac *S. aureus* Genotyping DNA microarray (Alere Technologies GmbH, Jena, Germany); 12 additional virulence genes were included (Supplementary Table S2).

Multilocus sequence types (MLST) were *in silico* extracted from the assembled genomes using the scheme by Enright et al. (2000).

### Cytotoxicity Essay

We performed cytotoxicity assays to test if statistical findings could be confirmed *in vitro*. Human polymorphonuclear neutrophils (PMN) were freshly isolated from sodium citrate-treated blood of healthy Caucasian voluntary donors. For isolation, dextran-sedimentation and density gradient centrifugation using Ficoll-Paque Plus (GE Healthcare, Solingen, Germany) was used according to the manufacturer’s instruction. Residual erythrocytes were eliminated by hypotonic lysis. PMNs were re-suspended in Dulbecco’s modified Eagle’s medium (Biochrom, Berlin, Germany) with 1% human serum albumin and immediately used for the experiments. Bacterial supernatants were prepared by growing bacteria in 10 ml of brain-heart infusion (BHI) broth in a rotary shaker (160 rpm) at 37°C for 15 h and pelleted for 10 min at 6800 g. Supernatants were sterile-filtered through a Filtropur S-filter unit (0.2 μm; Sarstedt, Nümbrecht, Germany) and used for the experiments.

For analysis of cell death induction, PMNs (5 × 10^6^ cells/ml) were incubated with bacteria culture supernatant for 30 min at room temperature, followed by staining with 10 μg/ml propidium iodide (Fluka, Taufkirchen, Germany). Controls were performed using 1–10% BHI instead of bacteria supernatants. Cytotoxicity was expressed as the proportion of propidium iodide positive cells (Löffler et al., 2010).

### Statistics

To assess the overall prevalence of the tested virulence factors, we first compared *S. aureus* from colonization and infection (*X*^2^-test or Fisher’s exact test, where appropriate). The virulence factors that had at least a weak evidence for association with clinical isolates (OR ≥ 1 and *p* ≤ 0.2) were selected to assess their association with major *S. aureus* infections. The impact of selected virulence factors on SSTI infection was modeled in a logistic regression analysis adjusted for potential confounders such as age, effective antimicrobial therapy and comorbidities. A therapy was considered to be effective if the respective antibiotic was tested “susceptible.” Monotherapies with aminoglycosides were not considered to be effective.

Normally distributed continuous variables were compared using student’s *t*-test.

The significance level was set at α = 0.05. Statistical analyses were done with “R” and the package epiDisplay.

## Results

In total, 100 *S. aureus* from asymptomatic carriers were included. Within the predefined study period of 2.5 years, we additionally recruited 88 patients with a community-associated infection. Of these, two patients were later excluded due to a non-*S. aureus* infection. Hence, 86 community-associated *S. aureus* were considered for the final analysis. Carriers and patients with a *S. aureus* infection were comparable in terms of age, sex, comorbidities and exposure to healthcare (i.e., hospitalization, **Table 1**). All participants (*n* = 186) denied a history of tuberculosis.

**Table 1:** Demographic characteristics of asymptomatic *Staphylococcus aureus* carriers and patients with a community-acquired *S. aureus* infection, DRC.

		*S. aureus*		OR (95% CI)	*P*-value
		Carriers (*n* = 100)	Infected patients (*n* = 86)		
Age, mean ±*SD*		23.6 ± 14.2	28.3 ± 20.1	NA	0.07
Sex, *n* (%) female		47 (47%)	39 (45%)	0.9 (0.5–1.7)	0.8
Hospitalization in the past^a^, *n* (%)		0 (0%)	0 (0%)	NA	NA
HIV infection, *n* (%)		3 (3%)	1 (1%)	0.4 (0.01–4.9)	0.6
McCabe classification	Non-fatal disease, *n* (%)	0 (0%)	2 (2%)	Inf (0.2–Inf)	0.2
	Ultimately fatal disease, *n* (%)	0 (0%)	1 (1%)	Inf (0.03–Inf)	0.5
	Rapidly fatal disease, *n* (%)	0 (0%)	1 (1%)	Inf (0.03–Inf)	0.5

*^a^Between 1 and 6 months prior to inclusion. NA, not applicable*.

Clinical *S. aureus* were isolated from skin and soft tissue infection (*n* = 57), urinary tract infection (*n* = 10), ear–eye–nose–throat infection (*n* = 7), bloodstream infection (*n* = 4) and others (*n* = 8). In general, comorbidities were low in patients with an *S. aureus* infection as mirrored by a median Charlson score of 0 (range: 0–2).

Antimicrobial resistance rates in isolates from colonization vs. infection were similar for penicillin (97% vs. 95.4%, *p* = 0.71), oxacillin (26% vs 33.7%, *p* = 0.25), clindamycin (4% vs. 3.5%, *p* = 1), erythromycin (21% vs. 30.2%, *p* = 0.15) and cotrimoxazole (62% vs. 59.3%, Supplementary Table S3). Higher resistance rates were found in isolates from infection compared to colonization for tetracycline (74.4% vs. 56%, *p* = 0.009), levofloxacin (29.1% vs. 16%, *p* = 0.032) and gentamicin (29.1% vs. 16%, *p* = 0.006, Supplementary Table S3). All isolates were susceptible to glycopeptides, linezolid and daptomycin.

On average, WGS of the 186 isolates resulted in a 96-fold (range: 38–175) coverage and in 231 contigs (85–561) with N50 value of 61748 (7845–171278). All 186 isolates were included in the final analysis after having passed our internal quality control ( ≥ 95% correctly identified cgMLST targets). From all WGS datasets, the MLST sequence types (ST) could be extracted. They were equally distributed among isolates from infection and colonization (*p* > 0.05, logistic regression). The most common STs (infection vs. colonization) were ST8 [43.0% (*n* = 37) vs. 32% (*n* = 32)], ST152 [22.1% (*n* = 19) vs. 21% (*n* = 21)], ST15 [6.8% (*n* = 5) vs. 10% (*n* = 10)] and ST121 [9.3% (*n* = 8) vs. 1% (*n* = 1)]. The predominant MRSA clones were ST8-MRSA-V/VII (t1476, t6940, t12377, t15642, t15643, PVL-negative, *n* = 40) followed by ST88-MRSA-IV (t690, t786, t4013, PVL-negative, *n* = 6), ST5-MRSA-IV (t002, t105, PVL-negative, *n* = 3), ST152-MRSA-II and -V/VII (t5691, t15644P, VL positive, *n* = 3) and ST6-MRSA (t2915, PVL-negative, *n* = 1), ST8-MRSA-V/VII (t1476, PVL positive, *n* = 1) and ST5-MRSA-IV (t311, PVL positive, *n* = 1).

All isolates were confirmed as *S. aureus* by the presence of *nuc* and *clfA*. All isolates from colonization and infection were positive for *aur*, *psmα*1–4, and *isdC* (see Supplementary Table S2 for details and function). If partially detected or potentially non-functional genes were included, all 186 isolates were positive for the following targets (proportion of cropped and failed targets): *ebpS* (0.5%), *eno* (0.5%), *hla* (9.7%), *icaA* (1.1%), *icaD* (1.6%), *clfB* (0.5%), *isaB* (1.6%), *isdB* (1.6%), *lgt* (0.5%), *sspA* (1.6%), *sspB* (1.6%), *saeS* (1.1%), and *vraS* (1.1%, Supplementary Table S2). The proportion of partially identified or “failed targets” are shown in Supplementary Table S2 for each virulence factor encoding gene.

Among the leukocidins, *lukG*/*lukH* was found in all clinical and nasal isolates; *lukD*/*lukE* was the second most prevalent leukocidin (68.3%, *n* = 127), followed by *lukS*-PV/*lukF*-PV (37.6%, *n* = 70, Supplementary Table S2). The presence of *lukS*-PV/*lukF*-PV was mainly associated with ST152 (55.7%, *n* = 39), ST121 (12.9%, *n* = 9) and ST15 (7.1%, *n* = 5).

Overall, the most abundant *lukS*-PV/*lukF*-PV variant was the R variant (61.4%, *n* = 43) followed by the H2 (28.6%, *n* = 20) and H1 (4.3%, *n* = 3) variant. Sequencing failure occurred in 4 cases (5.7%). Noteworthy, the R variant was more frequently found in SSTI (36.8%, *n* = 21) compared to other infection (10.3%, *n* = 3, OR = 5.1, 95% CI: 1.4–18.7, *p* = 0.01).

The majority of isolates carried *hla* (90.3%, *n* = 168) and *hld* (91.4%, *n* = 170, Supplementary Table S2). Non-truncated *hlb* was only detected in 22% (*n* = 41) of all isolates and was associated with ST152 (85.4%, *n* = 35) and ST8 (7.3%, *n* = 3). In these *hlb* positive isolates, *sak* (90%, *n* = 37), *scn* (81%, *n* = 33), *sea*/*sep* (2%, *n* = 1) and *chp* (5%, *n* = 2) were also detected.

The complete *hlg* operon comprising *hlgA*, *hlgB* and *hlgC* was detected in 79.6% (*n* = 148) of all isolates. All isolates with a deletion of *hlgC* (20.4%, *n* = 38) belonged to ST152.

The virulence factors *see*, *lukM*/*lukF*-PV83, *cap1*, *etB*, *edinC*, *bap* and *sasX* were not detected.

Among all virulence factors, only *agrIV*, *agr* deficiency, *seb*, *lukF*-PV/*lukS*-PV, *hlb*, *adsA* and *tst1* showed at least a weak association with infection (*p* ≤ 0.2 and OR ≥ 1, Supplementary Table S2) and were included in the multivariate analysis to assess their association with SSTI. Other entities were not analyzed due to a small sample size (**Table 2**). SSTI (*n* = 57) comprised abscess (*n* = 37), wound infection (*n* = 9), furuncle (*n* = 4), panaritium (*n* = 3), pyomyositis (*n* = 2), and others (*n* = 2).

**Table 2:** Association of *S. aureus* virulence factors with community-acquired skin and soft tissue infection (SSTI).

Virulence factor (*n*)	SSTI (*n* = 57),	Other infections	OR (95% CI)	*P*-value
	*n* (%)	(*n* = 29)^a^, *n* (%)		
*agrIV* (9)	8 (14%)	1 (4%)	4.6 (0.5–38.5)	0.2
*agr* deficiency (6)	5 (9%)	1 (4%)	2.7 (0.3–24.2)	0.4
*seb* (10)	8 (14%)	2 (7%)	2.2 (0.4–11.1)	0.3
*lukF*-PV/*lukS*-PV (37)	31 (54%)	6 (21%)	4.6 (1.6–12.9)	0.004
*hlb* (23)	20 (35%)	3 (10%)	4.7 (1.3–17.4)	0.02
*adsA* (84)	56 (98%)	28 (97%)	2 (0.1–33.2)	0.6
*tst1* (25)	16 (28%)	9 (31%)	0.9 (0.3–2.3)	0.8

*Only virulence factors were included in this multivariate analysis if they showed at least weak evidence for an association with infection (p ≤ 0.2 and OR ≥ 1), see Supplementary Table S2. ^a^ Including urinary tract infection (n = 10), ENT infection (n = 7), bloodstream (n = 4), respiratory tract infection (n = 2), amnionitis, eye infection, foreign body infection, joint infection, peritonitis, prostatitis (each n = 1).*

Both *lukS*-PV/*lukF*-PV and *hlb* were significantly associated with SSTI (*p* ≤ 0.02, **Table 2**). They were also frequently co-detected (OR = 11.9, 95% CI: 3.6–39.8, *p* < 0.0001): 22% (*n* = 19) of isolates from infection harbored both toxins.

Next, we analyzed the impact of *lukS*-PV/*lukF*-PV and *hlb* on the course of SSTI. Since the impact of virulence factors on clinical infection can be confounded by host factors, we adjusted the logistic regression analysis for age, an effective antimicrobial therapy and for comorbidities using the Charlson comorbidity score.

Isolates from SSTI carrying *lukS*-PV/*lukF*-PV (*n* = 37), *hlb* (*n* = 23) or both (*n* = 19) were significantly associated with abscesses/furuncles and recurrent SSTI (*p* < 0.02, **Table 3**). Of the 57 patients with SSTI, only 5 underwent surgery (i.e., incision, drainage, 9%), no patient died during a follow up of 14 days. There was no evidence for an association of *lukS*-PV/*lukF*-PV and/or *hlb* with medical interventions (i.e., incision, drainage, removal of foreign bodies).

**Table 3:** Impact of Panton-Valentine leukocidin (PVL) (*lukF*-PV/*lukS*-PV) and β-haemolysin (*hlb*) on SSTI.

Characteristics (*n*)	*lukF*-PV/*lukS*-PV			*hlb* positive (*n* = 23)			*lukF*-PV/*lukS*-PV and *hlb*		
	positive (*n* = 37)						positive (*n* = 19)		
	*N*	OR (95% CI)	*P*-value	*N*	OR (95% CI)	*P*-value	*N*	OR (95% CI)	*P*-value
Abscess and furuncles (46)	28	4.9 (1.8–13.3)	0.002	18	4.1 (1.3–12.7)	0.02	16	5.7 (1.5–21.7)	0.01
Recurrent infection (12)	10	7.7 (1.5–39.7)	0.02	7	4.6 (1.2–17)	0.02	7	6.26 (1.63,23.99)	0.007

*OR (95% CI) and P-value are adjusted for age, comorbidities (Charlson comorbidity score) and effective antimicrobial therapy on day 1.*

As the association with abscesses and furuncles was strongest if isolates carried both *lukS*-PV/*lukF*-PV and *hlb* (OR = 5.7, 95% CI: 1.5–21.7, *p* = 0.01), we wondered if a synergistic effect of PVL and Hlb can be detected *in vitro*. We therefore performed a cytotoxicity assay on supernatants of *lukS*-PV/*lukF*-PV-positive isolates with or without *hlb*. Clinical isolates were randomly selected for each group. Cytotoxicity of supernatants increased in a dose-dependent manner but was similar in each group (**Supplementary Figure S1**).

## Discussion

We analyzed the impact of the *S. aureus* virulome on the clinical course of infection using WGS. The main finding is a significant association of (recurrent) SSTI with *lukS*-PV/*lukF*-PV and/or *hlb* positive *S. aureus*. This fuels the controversial discussion on the role of PVL and highlights the need to study the impact of hemolysins on *S. aureus* infection particularly in PVL-endemic regions in Africa.

The high proportion of SSTI and the low comorbidity score in our cohort study reflects the typical spectrum of disease and patient population of community acquired infections (Naimi et al., 2003). The high proportion of MRSA among isolates from colonization (26%) and infection (34%, Supplementary Table S3) is striking. High MRSA rates (15.9%) have also been reported from Kisangani (Eastern DRC) (De Boeck et al., 2015).

Although all isolates passed the internal quality control ( ≥ 95% correctly identified cgMLST targets) some virulence genes of the core genome (i.e., *hla*, *hld*, *icaC*, *splA*, *splB*) were not detected in some isolates (Supplementary Table S2). This might be due to true variations in the core genome or is related to technical issues. The latter, however, is unlikely, as the same bioinformatics algorithm has been applied and successfully validated with DNA microarray data (Strauss et al., 2016).

Among the 79 virulence factors, only *lukS*-PV/*lukF*-PV and *hlb* were significantly associated with SSTI (**Tables 2**, **3**). While an association of SSTI with PVL has been shown already in PVL-endemic areas in Africa, our results also suggest an additional role of Hlb on the development of SSTI in PVL-endemic areas (Schaumburg et al., 2011).

We were unable to detect an impact of PVL on the frequency of medical interventions (i.e., incision of abscesses, drainage). This is in contrast to a study on travelers with SSTI, where PVL was a risk factor for hospital admission and surgical drainage (Nurjadi et al., 2015).

It is possible that the association of PVL with SSTI could be confounded by Hlb since both virulence factors were co-detected in 22% of clinical isolates. Our study suggests that this co-detection does not affect cytotoxicity as the proportion of dead cells was similar after incubation of supernatants of *lukS*-PV/*lukF*-PV-positive *S. aureus* with or without *hlb* (**Supplementary Figure S1**). This could be explained by a functional redundancy of leukocidins and Hlb to lyse leukocytes. In future studies, both PVL and Hlb should be taken into account when assessing the mechanism of SSTI in Africa. Hlb was mainly detected in ST152 isolates, consistent with findings from the Gambia (Senghore et al., 2016). The clonal complex CC152 is wide spread in Africa (8–21% of all MSSA) (Ruimy et al., 2008; Breurec et al., 2011). Hlb might therefore contribute to *S. aureus* pathogenicity more significantly in Africa than elsewhere.

Noteworthy, we co-detected non-truncated *hlb* with virulence genes (*sak*, *sea*/*sep*, *scn*, *chp*) that are usually encoded by *hlb*-converting phages (Kumagai et al., 2007). This suggests that these phages could have been translocated to atypical insertion sites. This seems to be more common in isolates from infection compared to colonization (Goerke et al., 2006).

The predominance of *lukS*-PV/*lukF*-PV variant R over H rather reflects the proportion in the United States (R: 96.7%, H: 3.3%) than in the rest of the world (e.g., South Africa, R: 5.6%, H: 94.4%) (O’Hara et al., 2008). Current evidence suggests that the H variant is the ancestor of the R variant. Although R variants are considered to be fitter than the H variant; both have a similar leukotoxicity (O’Hara et al., 2008; Besseyre des Horts et al., 2010).

Some limitations of our study need to be addressed. First, the sample size of clinical isolates was small and we might have detected only those virulence factors that exhibit strong association with SSTI. Second, we did not quantify the amount of secreted PVL and Hlb in the supernatant in the cytotoxicity assay and might have also detected the cytolytic activity of other leukocidins (e.g., HlgCB, lukDE, lukGH). Third, the sole presence of a virulence gene does not guarantee its expression. The impact of the virulome on bacterial pathogenesis should be therefore assessed with caution. Fourth, the non-detection of genes does not necessarily prove their biological absence, but may also be caused by technical limitations of shotgun sequencing and *de novo* assembly. However, the technical error rate of WGS was shown to be < 2% (Strauss et al., 2016).

## Conclusion

Our study strongly suggests that not only PVL but also Hlb significantly contributes to the development and recurrence of *S. aureus* SSTI in PVL-endemic African countries.

## Author Contributions

DM, GP, J-JM-T, and FS designed the study, ML, PP, and FS recruited participants and performed culture based microbiological analyses. LS and AM performed whole genome sequencing. SN performed functional assays. LS and FS analyzed the data. ML, PP, LS, SN, and FS drafted the manuscript. AM, DM, J-JM-T, and GP revised the manuscript.

## Conflict of Interest Statement

The authors declare that the research was conducted in the absence of any commercial or financial relationships that could be construed as a potential conflict of interest.

## Footnotes

1 https://www.ebi.ac.uk/ena

## Supplementary material

FIGURE S1 Cytotoxicity of representative PVL- and Hlb-positive *S. aureus* from the Democratic Republic of the Congo. Fresh human polymorphonuclear neutrophils (PMN) were incubated with different concentrations of supernatants (color coded, in %) of *S. aureus* overnight cultures. The proportions of propidium iodide (PI) stained (dead) cells were quantified using flow cytometry. Each group consists of three randomly selected clinical isolates. The mean value (+SD) derived from three independent experiments. A further statistical analysis was not done due to a small sample size.
