# Supplementary material for: The Impact of the Staphylococcus aureus Virulome on Infection in a Developing Country: A Cohort Study
Source: Front Microbiol. 2017 Aug 29;8:1662. doi: 10.3389/fmicb.2017.01662 (PMC5581934; doi:10.3389/fmicb.2017.01662)
Supplement: Supplementary file 3 [file Table_2.docx]

Table S2: Comparison of virulence factors and genotypes of *S. aureus* from asymptomatic carriers and patients with a community-acquired *S. aureus* infection, Democratic Republic of the Congo

|  | Virulence factors gene | Virulence factor | *S. aureus,* n (%) | | | | OR (95% CI) | p-value |
| --- | --- | --- | --- | --- | --- | --- | --- | --- |
|  |  |  | Total, (n=186) | | Carriers (n=100) | Infected patients (n=86) |  |  |
|  |  |  | intact genes | cropped/failed targets^a^ |  |  |  |  |
| Regulation | *agrI* | accessory gene regulator I | 124 (66.7) | NA^b^ | 69 (69) | 55 (64) | 0.8 (0.4-1.5) | 0.5 |
|  | *agrII* | accessory gene regulator II | 27 (14.5) | NA^b^ | 18 (18) | 9 (10.5) | 0.5 (0.2-1.3) | 0.2 |
|  | *agrIII* | accessory gene regulator III | 18 (9.6) | NA^b^ | 11 (11) | 7 (8.1) | 0.7 (0.3-1.9) | 0.5 |
|  | *agrIV* | accessory gene regulator IV | 10 (5.4) | NA^b^ | 1 (1) | 9 (10.5) | 11.6 (1.4-93.3) | **0.006** |
|  | *agr* deficiency | - | 7 (3.8) | NA^b^ | 1 (1) | 6 (7) | 7.4 (0.9-63.0) | **0.05** |
|  | *sarA* | accessory regulator A | 180 (96.8) | 2 (1.1) | 98 (98) | 82 (95.4) | 0.4 (0.1- 2.3) | 0.4 |
|  | *saeS* | histidine protein kinase | 184 (98.9) | 2 (1.1) | 100 (100) | 84 (97.7) | NA | 0.2 |
|  | *vraS* | sensor protein | 185 (99.5) | 1 (0.5) | 99 (99) | 86 (100) | NA | 1 |
| Enterotoxins | *sea-sep* | enterotoxin A-P | 25 (13.4) | 0 (0) | 16 (16) | 9 (10.5) | 0.6 (0.3-1.5) | 0.3 |
|  | *seb* | enterotoxin B | 15 (8.1) | 1 (0.5) | 5 (5) | 10 (11.6) | 2.5 (0.8-7.6) | **0.1** |
|  | *sec* | enterotoxin C | 6 (3.2) | 2 (1.1) | 4 (4) | 2 (2.3) | 0.6 (0.1-3.2) | 0.7 |
|  | *sed* | enterotoxin D | 1 (0.5) | 0 (0) | 1 (1) | 0 (0) | NA | 1 |
|  | *see* | enterotoxin E | 0 (0) | 0 (0) | 0 (0) | 0 (0) | - | - |
|  | *seg* | enterotoxin G | 42 (22.6) | 0 (0) | 23 (23) | 19 (22.1) | 1.0 (0.5-1.9) | 0.9 |
|  | *seh* | enterotoxin H | 7 (3.8) | 0 (0) | 2 (2) | 5 (5.8) | 3.0 (0.6-16) | 0.3 |
|  | *sei* | enterotoxin I | 43 (23.1) | 2.1 (4) | 24 (24) | 19 (22.1) | 0.9 (0.5-1.8) | 0.8 |
|  | *sej* | enterotoxin J | 57 (30.6) | 0 (0) | 29 (29) | 28 (32.6) | 1.2 (0.6-2.2) | 0.6 |
|  | *sek* | enterotoxin K | 21 (11.3) | 3 (1.6) | 10 (10) | 11 (12.8) | 1.3 (0.5-3.3) | 0.6 |
|  | *sel* | enterotoxin L | 4 (2.2) | 1 (0.5) | 4 (4) | 0 (0) | NA | 0.1 |
|  | *sem* | enterotoxin M | 42 (22.6) | 1 (0.5) | 24 (24) | 18 (20.9) | 0.8 (0.4-1.7) | 0.6 |
|  | *sen* | enterotoxin N | 43 (23.1) | 3 (1.6) | 24 (24) | 19 (22.1) | 0.9 (0.5-1.8) | 0.8 |
|  | *seo* | enterotoxin O | 43 (23.1) | 2.1 (4) | 24 (24) | 19 (22.1) | 0.9 (0.5-1.8) | 0.8 |
|  | *ser* | enterotoxin R | 57 (30.6) | 1 (0.5) | 29 (29) | 28 (32.6) | 1.2 (0.6-2.2) | 0.6 |
|  | *seu* | enterotoxin U | 29 (15.6) | 3 (1.6) | 21 (21) | 8 (9.3) | 0.4 (0.2-0.9) | 0.03 |
| Leukocidins | *lukF-PV/lukS-PV* | Panton-Valentine leukocidin F/S | 70 (37.6) | NA^b^ | 33 (33) | 37 (43) | 1.5 (0.8-2.8) | **0.2** |
|  | *lukM/lukF-PV83* | Panton-Valentine leukocidin M/F | 0 (0) | NA^b^ | 0 (0) | 0 (0) | - | - |
|  | *lukD/lukE* | leukocidin D/E | 127 (68.3) | NA^b^ | 67 (67) | 60 (69.8) | 1.1 (0.6-2.1) | 0.7 |
|  | *lukG/lukH* | leukocidin G/H | 186 (100) | NA^b^ | 100 (100) | 86 (100) | - | - |
| Hemolysins | *hla* | hemolysin α | 168 (90.3) | 18 (9.7) | 97 (97) | 71 (82.6) | 0.2 (0.04-0.5) | 0.001 |
|  | *hlb* | hemolysin β | 41 (22) | 8 (4.3) | 18 (18) | 23 (26.7) | 1.7 (0.8-3.3) | **0.2** |
|  | *hlgABC* | hemolysin γ, component A, B and B | 148 (79.6) | NA^b^ | 80 (80) | 68 (79.1) | 0.94 (0.5-1.9) | 0.9 |
|  | *hlgAB* | hemolysin γ, component A and B | 38 (20.4) | NA^b^ | 20 (20) | 18 (20.9) | 1.1 (0.5-2) | 0.9 |
|  | *hld* | hemolysin δ | 170 (91.4) | 11 (5.9) | 97 (97) | 73 (84.9) | 0.2 (0.1-0.6) | 0.004 |
| Immune evasion factors | *sak* | staphylokinase | 164 (88.2) | 0 (0) | 88 (88) | 76 (88.4) | 1 (0.4-2.5) | 0.9 |
|  | *chp* | chemotaxis-inhibiting protein | 114 (61.3) | 3 (1.6) | 68 (68) | 46 (53.5) | 0.5 (0.3-1) | 0.04 |
|  | *scn* | complement inhibitor | 170 (91.4) | 1 (0.5) | 95 (95) | 75 (87.2) | 0.4 (0.1-1.1) | 0.06 |
|  | *adsA* | adenosin synthase | 175 (94.1) | 1 (0.5) | 91 (91) | 84 (97.7) | 4.2 (0.9-19.8) | **0.07** |
|  | *flipr* | formyl peptide receptor-like 1 sinhibitor | 162 (87.1) | 8 (4.3) | 85 (85) | 77 (89.5) | 1.5 (0.6-3.7) | 0.4 |
| Capsule | *cap 1* | capsule type 1 | 0 (0) | NA^b^ | 0 (0) | 0 (0) | - | - |
|  | *cap 5* | capsule type 5 | 128 (68.8) | NA^b^ | 66 (66) | 62 (72.1) | 1.3 (0.7-2.5) | 0.4 |
|  | *cap 8* | capsule type 8 | 58 (31.2) | NA^b^ | 34 (34) | 24 (27.9) | 0.8 (0.4-1.4) | 0.4 |
| Exfoliative toxins | *etA* | exfoliative toxin A | 5 (2.7) | 0 (0) | 5 (5) | 0 (0) | NA | 0.06 |
|  | *etB* | exfoliative toxin B | 0 (0) | 0 (0) | 0 (0) | 0 (0) | - | - |
|  | *etD* | exfoliative toxin D | 4 (2.2) | 0 (0) | 3 (3) | 1 (1.2) | 0.4 (0.04- 3.7) | 0.6 |
| Epithelial differentiation inhibitors | *edinA* | epithelial differentiation inhibitor A | 3 (1.6) | 0 (0) | 1 (1) | 2 (2.3) | 2.4 (0.2-26.5) | 0.6 |
|  | *edinB* | epithelial differentiation inhibitor B | 44 (23.7) | 0 (0) | 24 (24) | 20 (23.3) | 1 (0.5-1.9) | 0.9 |
|  | *edinC* | epithelial differentiation inhibitor C | 0 (0) | 0 (0) | 0 (0) | 0 (0) | - | - |
| Proteases | *aur* | aureolysin | 186 (100) | 0 (0) | 100 (100) | 86 (100) | - | - |
|  | *splA* | serinprotease A | 116 (62.4) | 3 (1.6) | 65 (65) | 51 (59.3) | 0.8 (0.4-1.4) | 0.4 |
|  | *splB* | serinprotease B | 125 (67.2) | 2 (1.1) | 67 (67) | 58 (67.4) | 1 (0.6-1.9) | 1 |
|  | *splE* | serinprotease E | 93 (50) | 4 (2.2) | 48 (48) | 45 (52.3) | 1.2 (0.7-2.1) | 0.6 |
|  | *sspA* | glutamylendopeptidase | 183 (98.4) | 3 (1.6) | 99 (99) | 84 (97.7) | 0.4 (0.04-4.8) | 0.6 |
|  | *sspB* | staphopain B | 183 (98.4) | 3 (1.6) | 100 (100) | 83 (96.5) | NA | 0.1 |
|  | *sspP* | staphopain A | 157 (84.4) | 29 (15.6) | 89 (89) | 68 (79.1) | 0.5 (0.2-1.1) | 0.06 |
| Surface proteins | *bap* | biofilm-associated surface protein | 0 (0) | 0 (0) | 0 (0) | 0 (0) | - | - |
|  | *cna* | collagen-binding adhesin | 76 (40.9) | 1 (0.5) | 38 (38) | 38 (44.2) | 1.3 (0.7-2.3) | 0.4 |
|  | *ebh* | cell wall associated fibronectin-binding protein | 186 (100) | 0 (0) | 100 (100) | 86 (100) | - | - |
|  | *ebpS* | elastin binding protein | 185 (99.5) | 1 (0.5) | 100 (100) | 85 (98.8) | 0 (0-NaN) | 0.5 |
|  | *eno* | enolase | 185 (99.5) | 1 (0.5) | 99 (99) | 86 (100) | NA | 1 |
|  | *eap* | extracellular adherence  protein | 141 (75.8) | 0 (0) | 77 (77) | 64 (74.4) |  |  |
|  | *fib* | fibrinogen binding protein | 139 (74.7) | 2 (1.1) | 77 (77) | 62 (72.1) | 0.9 (0.4-1.7) | 0.7 |
|  | *icaA* | intercellular adhesion protein A | 184 (98.9) | 2 (1.1) | 98 (98) | 86 (100) | NA | 0.5 |
|  | *icaC* | intercellular adhesion protein C | 175 (94.1) | 10 (5.4) | 97 (97) | 78 (90.7) | 0.3 (0.1-1.2) | 0.1 |
|  | *icaD* | intercellular adhesion protein D | 183 (98.4) | 3 (1.6) | 99 (99) | 84 (97.7) | 0.4 (0.04-4.8) | 0.6 |
|  | *sasG* | surface protein G | 113 (60.8) | 1 (0.5) | 63 (63) | 50 (58.1) | 0.8 (0.5-1.5) | 0.5 |
|  | *sasX* | surface protein X | 0 (0) | 0 (0) | 0 (0) | 0 (0) | - | - |
|  | *sdrC* | bone sialoprotein-binding protein C | 143 (76.9) | 1 (0.5) | 78 (78) | 65 (75.6) | 0.9 (0.4-1.7) | 0.7 |
|  | *sdrD* | bone sialoprotein-binding protein D | 174 (93.6) | 0 (0) | 96 (96) | 78 (90.7) | 0.4 (0.1-1.4) | 0.2 |
|  | *vwb* | van Willebrand factor binding protein | 184 (98.9) | 0 (0) | 100 (100) | 84 (97.7) | NA | 0.2 |
| Other | *tst1* | toxic shock syndrome toxin | 45 (24.2) | 1 (0.5) | 20 (20) | 25 (29.1) | 1.6 (0.8-3.2) | **0.2** |
|  | ACME locus | arginine catabolic mobile element | 3 (1.6) | NA^b^ | 1 (1) | 2 (2.3) | 2.4 (0.2-26.5) | 0.6 |
|  | *speG* | spermidine-acetyltransferase | 1 (0.5) | 0 (0) | 1 (1) | 0 (0) | NA | 1 |
|  | *sigS* | sigmafactor S | 173 (93.0) | 9 (4.8) | 98 (98) | 75 (87.2) | 0.1 (0.03-0.7) | 0.007 |
|  | *psmα1-4* | phenol-soluble modulin, α type | 186 (100) | NA^b^ | 100 (100) | 86 (100) | - | - |
|  | *psmβ1* | phenol-soluble modulin, β1 type | 185 (99.5) | 0 (0) | 100 (100) | 85 (98.8) | NA | 0.5 |
|  | *psmβ2* | phenol-soluble modulin, β2 type | 180 (96.8) | 0 (0) | 97 (97) | 83 (96.5) | 0.9 (0.2-4.4) | 1 |
|  | *isdA* | Iron surface determinant A | 182 (97.9) | 3 (1.6) | 99 (99) | 83 (96.5) | 0.3 (0.03-2.7) | 0.3 |
|  | *isdB* | Iron surface determinant B | 183 (98.4) | 3 (1.6) | 98 (98) | 85 (98.8) | 1.7 (0.2-19.5) | 1 |
|  | *isdC* | Iron surface determinant C | 186 (100) | 0 (0) | 100 (100) | 86 (100) | - | - |
|  | *lgt* | Lipoprotein  diacyl-glyceride  transferase | 185 (99.5) | 1 (0.5) | 99 (99) | 86 (100) | NA | 1 |

Note: NA (not applicable), p-values of virulence factors included in the multivariate analysis are in bold.

^a^Targets that were partially identified on a cropped contig or “failed targets” (i.e. containing internal stop codons, frame shifts, or nucleotide ambiguities)

^b^Cropped and failed targets were considered to be intact if they were found within operons/prophages and if the other genes of the respective operon/prophage were present
