# Supplementary material for: The Impact of the Staphylococcus aureus Virulome on Infection in a Developing Country: A Cohort Study
Source: Front Microbiol. 2017 Aug 29;8:1662. doi: 10.3389/fmicb.2017.01662 (PMC5581934; doi:10.3389/fmicb.2017.01662)
Supplement: Supplementary file 4 [file Table_3.DOCX]

Table S3: Comparison of antimicrobial resistance of *S. aureus* from asymptomatic carriers and patients with a community-acquired *S. aureus* infection, Democratic Republic of the Congo

| Antibiotic | Resistant *S. aureus,* n (%) | | | OR (95% CI) | p-value |
| --- | --- | --- | --- | --- | --- |
|  | Total (n=186) | Carriers (n=100) | Infected patients (n=86) |  |  |
| Penicillin | 179 (96.2%) | 97 (97%) | 82 (95%) | 1.6 (0.3-11.1) | 0.7 |
| Oxacillin | 55 (29.6%) | 26 (26%) | 29 (34%) | 0.7 (0.4-1.4) | 0.3 |
| Clindamycin | 7 (3.8%) | 4 (4%) | 3 (4%) | 1.2 (0.2- 8.1) | 1 |
| Erythromycin | 47 (25.3%) | 21 (21%) | 26 (30%) | 0.6 (0.3-1.3) | 0.2 |
| Tetracycline | 120 (64.5%) | 56 (56%) | 64 (74%) | 0.4 (0.2-0.9) | 0.009 |
| Levofloxacin | 41 (22.0%) | 16 (16%) | 25 (29%) | 0.5 (0.2-1) | 0.03 |
| Gentamicin | 63 (33.9%) | 25 (25%) | 38 (44%) | 0.4 (0.2-0.8) | 0.006 |
| Cotrimoxazole | 113 (60.8%) | 62 (62%) | 51 (59%) | 1.1 (0.6-2.1) | 0.7 |
